# Editorial: Multimodal interventions in Alzheimer's disease: from basic research to clinical practice

**DOI:** 10.3389/fneur.2023.1303733

**Published:** 2023-10-20

**Authors:** Tongtong Zhang, Lin Cong, Ben Chen, Zhuohao He, Guozhong Li, Qi Qin

**Affiliations:** ^1^Innovation Center for Neurological Disorders, Department of Neurology, Xuanwu Hospital, Capital Medical University, National Center for Neurological Disorders, Beijing, China; ^2^Department of Neurology, Shandong Provincial Hospital Affiliated to Shandong First Medical University, Jinan, Shandong, China; ^3^Center for Geriatric Neuroscience, The Affiliated Brain Hospital of Guangzhou Medical University, Guangzhou, Guangdong, China; ^4^Shanghai Institute of Organic Chemistry, Chinese Academy of Sciences (CAS), Shanghai, China; ^5^Department of Neurology, The First Affiliated Hospital of Harbin Medical University, Harbin, Heilongjiang, China

**Keywords:** Alzheimer's disease, basic research, clinical practice, multimodal interventions, treatment

By 2050, it is projected that Alzheimer's disease (AD) will remain the prevailing variant of dementia, affecting an estimated 131.5 million people (1). AD onset is irreversible despite significant efforts to combat this condition. AD and its related pathological changes are unavoidable and incurable and may occur as early as 20 to 30 years before the onset of dementia. Consequently, taking action to address various risk elements in elderly individuals without dementia and even those in middle age could potentially stave off or postpone the inception of AD. These factors encompass a broad spectrum, spanning psychosocial elements, pre-existing health conditions, lifestyle choices, and more (2). In recent years, non-pharmacological treatments for AD have garnered increasing attention as supplementary measures. Evidence demonstrates that non-pharmacological interventions, including physical therapy, acupuncture, exercise therapy, cognitive training, and psychotherapy, yield positive impacts on the cognitive function of AD patients and can substantially enhance their overall quality of life (3–5). In essence, the management of AD should encompass a holistic evaluation of diverse risk factors, disease stages, and pathogenic mechanisms. Non-pharmacological interventions should either complement pharmacological treatment, or be combined in various non-pharmacological treatment modalities, collectively elevating the cognitive functioning of AD patients. This multifaceted approach holds promise for achieving superior treatment outcomes in AD.

We aim to introduce multimodal diagnostic approaches and interventions in AD. While additional research is essential to gain a deeper comprehension of AD's pathogenesis of AD, delving into the assessment of risk factors for the disease and examining the influence of multimodal interventions on AD can potentially identify innovative avenues for mitigating risk.

Traditional beliefs prevalent in impoverished regions pose significant challenges in diagnosing dementia. A comprehensive review undertaken by Adebisi and Salawu investigated the influence of these traditional beliefs in Sub-Saharan Africa (SSA) on individuals with dementia-related disorders. This influence manifests in various ways, including the absence of adequate care and support, social stigma, public mistreatment, and the imposition of punitive measures. The review specifically examined two prevalent beliefs: attributing dementia to evil spirit possession and associating it with witchcraft and bewitchment. The findings underscore the pressing need for initiatives focused on education, awareness, and providing appropriate care and support to address these challenges and enhance the wellbeing of those affected by dementia. This article sheds light on the fact that factors such as low educational level and psychosocial elements are potentially modifiable risk factors for AD. Implementing corresponding interventions can effectively reduce the incidence of AD in underserved areas.

Regarding treatment, the ketogenic diet is a dietary regimen characterized by low carbohydrate intake and high fat consumption. This dietary approach induces a state of ketosis in the body, wherein ketone bodies become the primary energy source (6, 7). Another theory posits that ketone-based medications exert both direct and epigenetic influences, thereby contributing to their anti-inflammatory, antioxidant, and neuroprotective properties, including their ability to combat amyloid-related issues (8). Bohnen et al. conducted an extensive literature review examining the efficacy of various ketogenic interventions in the context of mild cognitive impairment, AD, and Parkinson's disease. Their findings revealed that therapeutic ketogenic trials have the potential to enhance cognitive function in patients with mild cognitive impairment and in individuals with mild to moderate AD who are negative for the apolipoprotein ε4 allele. Although the specific methodologies of these ketogenic interventions differed among the various study protocols, they have positive effect on cognition. Future studies should delineate optimal utilization of therapeutic ketosis via a precision medicine approach and optimize the effectiveness of ketogenic interventions by lifestyle factors.

As AD advances, patients become susceptible to a spectrum of complications that accelerate disease progression and impose greater burdens on caregivers. Furthermore, the incidence of readmissions among elderly AD patients significantly rises, leading to an elevated in-hospital mortality rate (8, 9). Despite the existence of several risk prediction models for AD, encompassing machine learning techniques and artificial intelligence, the availability of mortality risk prediction models tailored specifically for hospitalized AD patients remains limited (10, 11). In this context, Yao et al. harnessed the comorbidities observed during hospitalization to construct a prognostic nomogram for AD. Among these conditions, cerebro-cardiovascular disease, diabetes, chronic kidney disease, and chronic obstructive pulmonary disease stand out as significant contributors to the heightened risk of in-hospital mortality in patients diagnosed with AD. Through the identification and targeted mitigation of these critical risk factors, healthcare providers have the potential to enhance the management and outcomes of elderly AD patients grappling with comorbidities during their hospitalization.

In recent years, numerous scientific investigations have validated the advantages of non-pharmacological treatments, with a specific focus on physical exercise, in the realm of AD therapy (12, 13). These treatments exhibit remarkable efficacy in enhancing the brain's regenerative capabilities and adaptability, consequently bolstering the cognitive and motor functions of individuals with AD (14). Additionally, exercise may contribute to increased peripheral blood circulation and the release of neurotrophic factors in the brain, further reducing the risk of AD development (8). It is important to note, however, that personalized exercise regimens and expert guidance are imperative to ensure the safety and appropriateness of these exercise programs. David et al. embarked on a 6-month intervention study centered on physical activity among individuals with AD. They employed three distinct methods to gauge physical activity in older participants with cognitive impairment. The outcomes revealed that fitness trackers and physical activity diaries displayed the highest stability and could furnish comparable estimates of physical activity in individuals with AD. These methods excel in quantifying the physical activity interventions for AD and offer a multi-faceted perspective for a comprehensive multimodal approach to AD intervention.

Publications featured in this Research Topic showcase a variety of multimodal diagnostic approaches and interventions for AD. These approaches leverage a range of technologies and practical tools for mortality risk prediction, physical activity monitoring, and evaluating ketogenic interventions. Ultimately, adopting a multimodal approach to AD intervention holds considerable importance. This approach is valuable because it encompasses a comprehensive, synergistic, and personalized strategy tailored to the intricate characteristics of the condition. By integrating diverse intervention methods, there is the potential to postpone the advancement of the disease, elevate cognitive functioning, enhance the overall quality of life, and deliver superior care to individuals with AD.

## Author contributions

TZ: Writing—original draft. LC: Writing—review and editing. BC: Writing—review and editing. ZH: Writing—review and editing. GL: Writing—review and editing. QQ: Writing—review and editing.
